# Pulmonary artery stiffness in chronic obstructive pulmonary disease (copd) - the mesa copd study

**DOI:** 10.1186/1532-429X-13-S1-P73

**Published:** 2011-02-02

**Authors:** Chia-Ying Liu, Rui Jiang, Stephen Dashnaw, Aditya Jain, Antoinette Gomes, James Carr, David A Bluemke, Joao Lima, Martin Prince, Graham Barr

**Affiliations:** 1 The Johns Hopkins Hospital, Baltimore, MD, USA; 2 Department of Epidemiology, Mailman School of Public Health, Columbia University, New York, NY, USA; 3 Department of Medicine, University of California, Los Angeles, Los Angeles, CA, USA; 4 Radiology, Northwestern University, Chicago, IL, USA; 5 Clinical Center and National Institute of Biomedical Imaging and Bioengineering, National Institutes of Health, Bethesda, MD, USA; 6 Department of Medicine, Weill Cornell Medical College, New York, NY, USA

## Introduction

Arterial stiffness had been proposed as the cause of sub-clinical left ventricular dysfunction in patients with COPD. Vascular studies in COPD patients were mainly assessed by carotid-femoral (aortic) pulse wave velocity using micromanometer applanation tonometry. The evaluation of pulmonary artery (PA) elasticity in COPD has not been assessed. This study seeks to evaluate indices of PA stiffness in patients with COPD. We hypothesized that patients with COPD would have increased pulmonary artery stiffness compared to healthy individuals. To test this we determine the pulmonary artery area change (distensibility or pulsatility in %) by cardiac magnetic resonance imaging (MRI) and related PA distensibility to the severity of COPD.

## Methods

MRI studies were performed using a 1.5T scanner (EXCITE, GE Healthcare) on 71 participants recruited to date into an ongoing nested case-control study of current and former smokers, age 58-79 years, free of clinical cardiovascular disease. COPD presence and severity was defined on post-bronchodilator spirometry by GOLD criteria (FEV1/FVC <70%, FEV1 % predicted, >80=mild, 50-80%=moderate, 30-50%=severe). To measure left ventricular function, the heart was imaged with MRI in short-axis orientation using a retrospectively gated steady-state free precession sequence. Phase-contrast images perpendicular to the pulmonary trunk were obtained using a segmented fast gradient echo sequence (TR/TE=7.5/3.1ms, slice thickness=8mm, matrix 256 x256, 2 to 4 segments, 30 reconstructed cardiac phases, velocity encoding 100cm/s) without breath hold. Pulsatility of the pulmonary vessels (in %) was measured from the largest systolic and the smallest diastolic areas derived from the magnitude images as: *100%x(maximum area-minimum area)/minimum area*.

## Results

Participants with COPD had increased PA stiffness compared to normals as shown in Table 1, although statistical significance was not attained in the sample recruited to date (P-trend across the categories of COPD severity, 0.2). Table 2 demonstrates the multivariate analysis of the difference of pulsatility between COPD severity compared to normal controls after adjusting for age, gender, race, height, weight, body mass index, smoking status and cigarette packs per year. Associations were of greater magnitude for the right and left PA.

**Table 1:** 

COPD Severity	N	PA Pulsatility % (SE)	p
Normal	35	14.0 (5.0)	-
Mild	16	13.1 (4.5)	0.56
Moderate	18	12.6 (3.7)	0.29
Severe	2	11.8 (3.)	0.51

**Table 2:** 

COPD Severity	N	PA Pulsatility % (SE)	p
Normal	35	0 (ref)	-
Mild	16	-1.15 (1.6)	0.47
Moderate	18	-1.41 (1.4)	0.32
Severe	2	-3.15 (3.4)	0.36

## Conclusions

Patients with COPD may have increased pulmonary artery stiffness measured by MRI PC imaging. Ongoing recruitment into this study is may confirm these preliminary findings.

